# Fiber Composition in Sows’ Diets Modifies *Clostridioides difficile* Colonization in Their Offspring

**DOI:** 10.1007/s00284-022-02848-y

**Published:** 2022-04-09

**Authors:** Łukasz Grześkowiak, Eva-Maria Saliu, Beatriz Martínez-Vallespín, Anna Grete Wessels, Klaus Männer, Wilfried Vahjen, Jürgen Zentek

**Affiliations:** Institute of Animal Nutrition, Freie Universität Berlin, Königin-Luise-Str. 49, 14195 Berlin, Germany

## Abstract

Dietary fiber has a potential to modulate the gut microbiota in sows. We hypothesized that a maternal diet rich in either high- or low-fermentable fiber during gestation and lactation influences *Clostridioides difficile* gut colonization in suckling piglets. Twenty sows were fed gestation and lactation diets enriched with either high-fermentable sugar beet pulp (SBP) or low-fermentable lignocellulose (LNC) fibers. *C. difficile*, toxin B (TcdB), fecal score, microbial abundance (16S-rDNA sequencing) and metabolites were measured in the feces from the sows and their piglets. *C. difficile* concentration was higher in piglets from the sows fed LNC than SBP along the study (*P* ≤ 0.05). Higher prevalence of *C. difficile* was noted in three-week-old piglets from sows fed LNC vs. SBP (45% vs. 0%, *P* = 0.001). TcdB prevalence was higher in six-day-old piglets from the sows fed LNC *vs*. SBP (60% vs. 17%, *P* = 0.009). In sows, fecal microbial metabolites were higher in SBP than LNC, while *C. difficile* concentration showed no difference. Higher microbial diversity Shannon index was noted in sows from SBP vs. LNC one week before parturition and at the parturition (*P* ≤ 0.05). Piglets from SBP *vs*. LNC tended to have higher microbial diversity Shannon index at two and three weeks of age. Diets enriched with high-fermentable fiber compared to low-fermentable fiber in sows reduced *C. difficile* colonization in their piglets. Susceptibility to colonization by *C. difficile* in neonatal piglets can be modulated by the sows’ diet, supporting the hypothesis of the early microbial programming in the offspring and the importance of the sow-piglet couple.

**Supplementary Information:**

The online version contains supplementary material available at 10.1007/s00284-022-02848-y.

## Introduction

The association between sow and offspring is a critical factor for the microbial and immune development. After parturition, the neonatal piglet is exposed to an avalanche of diverse bacteria, which gradually populate the body surfaces including the gastrointestinal tract [1, 2]. Similar to humans, events taking place during gestation and after parturition may have an impact on the gut microecosystem in the offspring and intestinal health later in life [3–5]. Disruption of the natural microbial colonization process or perturbances of the intestinal ecosystem may enhance the susceptibility to gastrointestinal infections [6, 7]. The aspect of the sow–piglet association and its effect on early “microbial programming” and resilience to pathogens is gaining more attention [8]. On the contrary, the microbial association between mother and infant has already been widely studied [9, 10].

Gut bacteria require specific conditions for proliferation and metabolism in the host. The “windows of opportunity” for certain gut pathogens may depend on the age of the host or pathogens may benefit from gut microbial and immune dysbiosis [11, 12]. *Clostridioides difficile* is one of the pioneer colonizers in neonatal piglets and it has also been documented as a major cause of enteritis outbreaks in these animals [13]. In suckling piglets, *C. difficile* and its toxins can often be detected in feces up to two weeks after birth [14]. Both are occasionally found in weaned piglets and adult pigs indicating that sows could be a significant carrier of virulent *C. difficile* for their offspring [2]. Similar to piglets, toxigenic *C. difficile* is found in feces of human neonates, however, without any harm to the healthy host. Besides a hypothesis related to a lack of toxin receptors on infant gut epithelium, the toxin-neutralizing antibodies and other bioactive compounds present in colostrum and breastmilk, as well as the developing microbial colonization resistance, may be responsible for protection from *C. difficile* infection (CDI) in healthy infants [15]. Reducing the load of *C. difficile* inoculum in neonatal piglets and infants would be a promising approach to control *C. difficile* colonization and dissemination in the environment.

Diet is a strong modulating factor with a long-lasting impact on gut microbial ecosystem in pigs [1, 16]. Both, gestation and lactation periods seem to be the most promising stages for dietary interventions in sows to influence the fitness in the offspring. By altering the sows’ microbiota through nutritional factors, it may be possible to influence the piglets’ microbial development and health [17, 18]. Indeed, in humans, numerous data demonstrate associations between maternal nutritional status and microbiota on the infant gut microbiota, immune development and to a potential link to *C. difficile* [5, 9, 19, 20]. However, little is known about the impact of diet on the microbial association between sow and offspring and the establishment of the gut microbiota early in life. In this context, dietary fiber is an attractive feed ingredient that can influence the physiology and health of the sows and their offspring [21]. The source of fiber (i.e. soluble/high fermentable or insoluble/slowly fermentable) may shape the intestinal ecosystem of the sow and therefore of the offspring in different ways [22]. The influence of various types of fiber in the diet for lactating and gestating sows on *C. difficile* colonization in the offspring has not been studied yet. However, this approach could be a promising way to control the microbial colonization and metabolic patterns in sows and offspring and thereby protect against *C. difficile* expansion in suckling piglets. Therefore, we hypothesized that a sow diet rich in high- or low-fermentable fiber during gestation and lactation differently affects colonization of *C. difficile* in their piglets.

## Material and Methods

### Diets

Experimental gestation and lactation diets were formulated according to national recommendations to meet the sows’ requirements for nutrients [23]. The isoenergetic and isonitrogeneous diets provided high inclusion percentage of high-fermentable fiber source in form of sugar beet pulp (SBP; inclusion rate: 15% sugar beet pulp) or high percentage inclusion of low-fermentable fiber source in form of lignocellulose (LNC; inclusion rate: 15% lignocellulose), as shown in Table 1.

**Table 1:** Ingredients and chemical composition of the experimental diets

	Gestation diet		Lactation diet	
Ingredients (g/kg feed)	SBP	LNC	SBP	LNC
Sugar beet pulp^*^	150	25	150	25
Arbocel^†^	30	150	30	150
Barley	610	553	338	275
Soybean meal [49% CP]	77	111	171	204
Wheat	80	80	200	200
Premix^◊^	12	12	12	12
Calcium carbonate	7	11	9	10
Monocalcium phosphate	14	1	21	25
Lysine HCl	5	5	1	1
Soy oil	13	41	66	96
Salt	1	1	1	1
Threonine	1	1.3	1	1
Methionine	0.2	0.2	-	0.1
Calculated metabolizable energy (MJ/kg)	11.4	11.4	13.0	13.0
Analyzed composition (g/kg fresh matter)				
Dry matter	899.5	902.4	918.0	917.5
Crude ash	46.0	43.7	65.0	61.2
Crude protein	138.9	138.8	165.6	157.2
Crude fat	30.4	42.6	80.5	99.1
Crude fiber	66.1	110.5	70.3	123.7
Neutral detergent fiber	163.4	225.9	180.1	230.0
Acid detergent fiber	85.9	143.9	79.3	125.4
Acid detergent lignin	9.6	33.5	10.9	34.2
Insoluble dietary fiber	188.4	245.1	187.6	244.2
Soluble dietary fiber	55.5	49.1	61.6	46.4
Total dietary fiber	243.8	294.2	249.2	290.6
Starch	400.0	355.8	345.7	289.1

*SBP* sugar beet pulp-enriched diet, *LNC* lignocellulose-enriched diet, *CP* crude protein

^*^SBP (containing approximately 78% of total and 41% of soluble NSP) [61]

^†^Arbocel® (containing approximately 65% of lignocellulose, J. Rettenmaier & Söhne GmbH & Co. KG, Rosenberg, Germany)

^◊^Mineral and vitamin premix (Spezialfutter Neuruppin GmbH, Neuruppin, Germany), containing per kg DM: 130 g Na (as NaCl), 55 g Mg (as MgO), 210 mg retinol, 3 mg vitamin D_3_, 8 g DL-α-tocopherol, 300 mg menadione, 250 mg thiamine, 250 mg riboflavine, 400 mg vitamin B_6_, 2 mg vitamin B_12_, 2.5 g nicotinic acid, 100 mg folic acid, 25 mg biotin, 1 g pantothenate, 80 g choline chloride, 5 g Fe (as FeCO_3_), 1 g Cu (as CuSO_4_), 5 g Zn (as ZnO), 6 g Mn (as MnO), 45 mg I (as CaI_2_O_6_), 35 mg Se (as Na_2_SeO_3_)

### Feed Analyses

Weende proximate analysis (ash, crude fiber, crude protein, ether extract), neutral detergent fiber, acid detergent fiber, lignin and starch were determined using standard procedures VDLUFA (Verband Deutscher Landwirtschaftlicher Untersuchungs- und Forschungsanstalten) (VDLUFA III 3.1, VDLUFA III 4.1.1, VDLUFA III 6.1.4, VDLUFA III 8.1, VDLUFA III 5.1.1, VDLUFA III 7.2.1) [24].

### Animals, Housing and Feeding

Twenty German Landrace sows (six multiparous, 14 gilts) were randomly allocated to the experimental feeding groups, with equal proportion of multiparous and gilt sows in each group. During gestation and lactation, the sows were kept without straw or other plant materials. Twenty gestating sows were kept in groups of 10 animals and housed spatially individually in farrowing pens one-week ante-partum until weaning. The farrowing occurred naturally without artificial induction. The sows were fed restrictively during gestation, while ad libitum during lactation. They were fed experimental gestation and lactation diets enriched with SBP (*n* = 10) or LNC (*n* = 10), as described above. Lactation diets were provided to sows three days after farrowing. Water was available to the animals ad libitum.

One week after farrowing, one sow from the SBP group and one sow from the LNC group were excluded from the trial due to post-farrowing complications, not related to the experimental diets.

Newborn piglets were balanced for sex and weight and four representative animals per sow (2 males, 2 females) were tagged and their fecal samples were collected during the suckling period. Suckling piglets were not provided with creep feed.

### Sampling

**Fig. 1:**
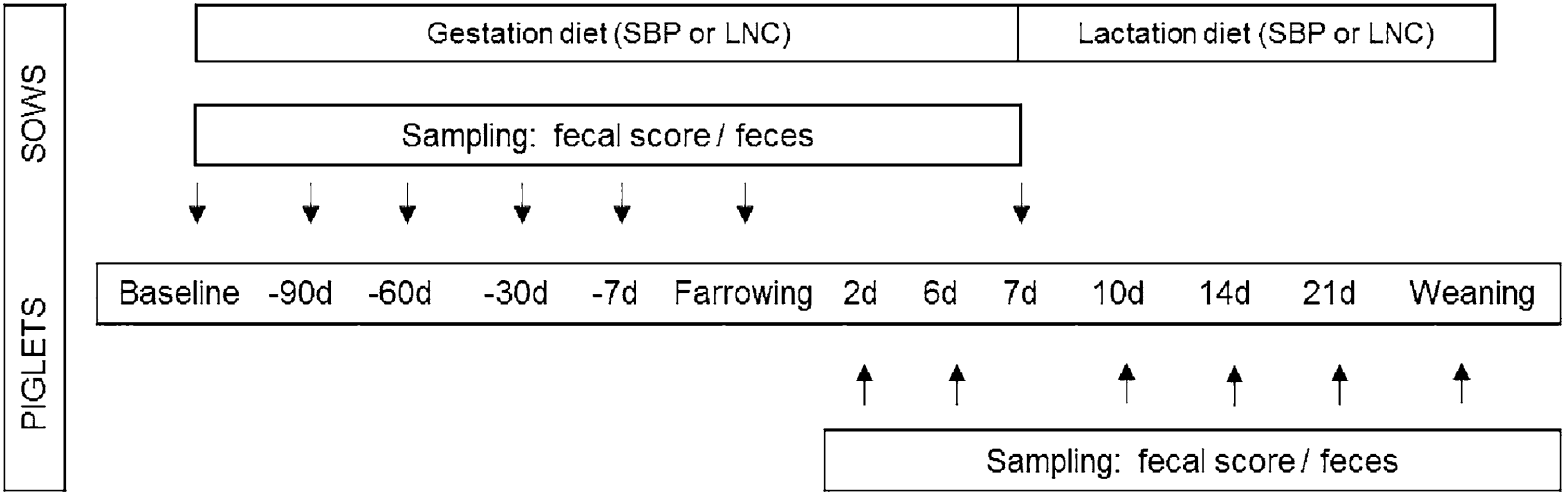
Experimental design including feeding and sampling of the sows and piglets. *SBP* sugar beet pulp-enriched diet, *LNC* lignocellulose-enriched diet, *d* day.

Fresh fecal samples were collected from all the sows seven days before farrowing, at farrowing and seven days after farrowing. Feces from piglets were collected at day two, six, 10, 14, 21 and at weaning. Fecal samples were stored frozen at − 30 °C until analyzed. Fecal scores of the sows and piglets were recorded throughout the trial. The seven-scale “Bristol stool form scale” was adapted to assess the fecal score for all sow and all piglet samples [25], in which an additional score (“0”) was included if meconium was present in piglet feces. The fecal score was as follows: 0, meconium; 1, separate and hard; 2, hard but lumpy; 3, soft with cracks; 4, soft and smooth; 5, soft blobs; 6, soft and mushy; 7, watery (diarrheic). The experimental design is illustrated in Fig. 1.

### Determination of *C. difficile* and Toxin B

Determination of *C. difficile* and toxin B in the feces was performed for all sow samples and for two of four piglets of each sow. *C. difficile* in the feces was quantified on *C. difficile*-ChromID selective agar plates (Biomerieux, France), as previously described [14].

To detect toxin B (TcdB) in the feces, enzyme-linked immunosorbent assay (ELISA) was carried out following the protocol of the ELISA commercial kit (tgcBIOMICS GmbH, Bingen, Germany) [14].

### DNA Extraction

Fecal samples selected randomly from four sows/group/time point and from one of their piglets were used for DNA extraction and bacterial characterization by 16S-rDNA sequencing. DNA from fecal samples (0.25 g) obtained from piglets was extracted using the NucleoSpin DNA Stool kit (Macherey–Nagel, Düren, Germany) and from sows was extracted using the QIAamp Power Fecal Pro DNA kit (Qiagen, Hilden, Germany) following the manufacturer’s instructions. The extraction protocols were preceded by repeated bead-beating on a FastPrep-24™ 5G homogenizer (MP Biomedicals, LLC, Santa Ana, California, USA) to increase DNA extraction efficiency from spore-forming [26] and likely also Gram-positive bacteria.

### Sequencing and Computational Data Analysis

DNA extracts were subjected to amplicon sequencing using an ﻿Illumina NextSeq500 sequencer (LGC, Berlin, Germany). Using a universal primer set, the 341F-785R V3-V4 region of the 16S rDNA was targeted and sequenced. The forward and reverse reads were combined using BBMerge tool (version 34.48) [27]. After demultiplexing, the resulting 16S-rDNA sequences of 108 samples were analyzed using QIIME2 pipeline [28] to determine microbial community profiles of each sample. Specifically, quality control was performed by DADA2 [29] routine within QIIME2. In the quality control process, chimeric sequences were removed, and regions of sequences with low-quality scores were truncated. The exact amplicon sequence variants (ASV) [30] and their respective counts in each sample were determined using DADA2. The ASV with total counts less than five sequences were excluded from further analysis to increase confidence of sequence reads. To account for the uneven distribution of sequences within samples, normalization was done through rarefication [31]. After rarefication to 10,000 reads, the sequence read depth of the samples was in saturation. Taxonomic assignment of the exact amplicon sequence variants was done using QIIME2’s feature classifier [32, 33] together with the SILVA SSU database [34] (release 132). Subsequent genus-level taxonomic profiles were generated based on the assignment of sequences and their corresponding counts. Bacterial taxa present in at least 10% of 108 samples were included in further statistical analysis on the relative abundances; they accounted for 172 taxa. Microbial diversity represented by Shannon index was calculated based on ASV using vegan package [35]. Shannon index was calculated from the following formula [36]: $H^{\prime} = - \sum\limits_{i = 1}^{R} {pi\,\ln \,pi}$, where *R* is the observed number of species and pi is the proportional abundance of species i. The sequences have been deposited in a public repository: BioProject ID: PRJNA803022.

### Analysis of Metabolites in Sow Feces

Feces were assessed for d- and l-lactate using high-performance liquid chromatography on an Agilent 1100 chromatograph, as previously described [37]. Ammonia in the feces was analyzed photometrically using the Berthelot reaction and extinction was measured using Tecan Sunrise™ microplate reader (Tecan Austria GmbH, Grödig, Austria). ﻿Fecal short-chain fatty acids (SCFA) were determined by gas chromatography on an Agilent 6890 gas chromatography system with flame ionization detector and autosampler (Agilent Technologies, Böblingen, Germany) [37]. Biogenic amines in the feces (putrescine, cadaverine, tyramine, histamine, spermidine and spermine) were analyzed with ion-exchange chromatography, as previously described [37].

### Statistical Analyses

The data for *C. difficile*, TcdB and metabolites were analyzed by Mann–Whitney *U* test. Fisher's Exact test was used to test the percentages of positive values for *C. difficile* and TcdB. Differences for microbial diversity Shannon index and microbial abundance (16S-rDNA) were calculated by Mann–Whitney *U* test. Correlations between the concentrations of *C. difficile* and TcdB were assessed using the Spearman's correlation analysis procedure. Significant differences were considered at *P* ≤ 0.05. Statistical analyses were performed using the software SPSS 27.0 (SPSS Inc., Chicago, IL). The beeswarm plot was generated in RAWGraphs 2.0 beta [38].

## Results

### Bacterial Communities and Diversity Indices in Sow Feces

In the individual sow samples, we found between 45 and 100 bacterial taxa. The total number of identified bacterial taxa was 172, of which the 26 dominant taxa were displayed in stacked bar plots (Supplementary Figure S1). Here, taxa of *Clostridium* sensu stricto 1, *Lactobacillus* spp.*, Terrisporobacter* spp.*, Romboutsia* spp.*,* and *Streptococcus* spp. predominated the gut microbiota of sows from both dietary groups throughout the sampling period. Sows fed LNC had a significantly higher abundance of *Terrisporobacter* spp. in their feces, as compared to sows fed SBP 30 days before farrowing (*P* = 0.029). In addition, in this same gestation period, there was a trend for a higher abundance of *Bifidobacterium* spp. in the feces of sows fed SBP vs. LNC (*P* = 0.057). One week before the farrowing, the abundance of sequences belonging to *Muribaculaceae* family was slightly increased in sows fed SBP vs. LNC (*P* = 0.057). At farrowing, the abundance of *Maribaculaceae* increased significantly (*P* = 0.029) whereas sequences belonging to *Ruminococcaceae* family (*P* = 0.057), *Clostridium* sensu stricto 1 (*P* = 0.057) showed a trend for an increase in abundance in sows fed SBP vs. LNC. On the contrary, the abundance of *Lactobacillus* spp. showed an increasing trend in sows fed LNC vs. SBP at the farrowing. One week after farrowing, the abundance of *Lactobacillus* spp. showed a continuous trend for an increase in sows fed LNC vs. SBP (*P* = 0.057).

At farrowing, Shannon index was significantly higher in sows from SBP vs. LNC group (3.8 ± 0.10 vs. 2.7 ± 0.34, *P* = 0.029, respectively).

### Fecal Consistency and *C. difficile* Shedding in Sows

Feces of the sows from both feeding groups had a physiological consistency (score 3) throughout the gestation and lactation periods and constipation or diarrhea was not observed. Feces of sows fed SBP diet were moister and darker in color than feces of sows fed LNC diet.

*C. difficile* was not detectable one week before farrowing in any of the sows. It was determined at the farrowing day in only one sow from SBP group (log_10_ 2.30 CFU/g) and in one sow from LNC group (log_10_ 2.00 CFU/g). *C. difficile* was detected seven days post-partum in all the sows from SBP group (median log_10_ 3.5 CFU/g, min–max 2.0–4.5) and in all the sows from LNC group (median log_10_ 3.2 CFU/g, min–max 2.6–4.5) (*P* = 0.258). TcdB was not detected in any of the sows during the periparturient period.

### Fecal Metabolite Patterns in Sow Feces

Results on fecal metabolite patterns in sows during periparturient period are shown in Table 2. One week before farrowing, levels of spermidine were significantly higher in feces of sows fed SBP, as compared to sows fed LNC (*P* < 0.001). On the farrowing day, significantly higher concentrations of fecal propionate (*P* = 0.033), i-butyrate (*P* < 0.001), ammonia (*P* = 0.043), cadaverine (*P* = 0.028) and spermine (*P* = 0.001) were detected in feces of sows fed SBP, as compared to sows fed LNC. One week after farrowing, a trend for higher levels of acetate (*P* = 0.053), propionate (*P* = 0.053), as well as significantly higher i-butyrate (*P* = 0.013), i-valerate (*P* = 0.043), ammonia (*P* = 0.008) and spermidine (*P* = 0.008) were found in feces of sows fed SBP, as compared to sows fed LNC.

**Table 2:** Microbial metabolites (µmol/g wet weight) in feces of the sows (*n* = 10/age/group) fed diets containing high-fermentable sugar beet pulp (SBP) or low-fermentable lignocellulose (LNC) fibers during gestation and lactation

	One week before farrowing			Farrowing			One week after farrowing		
	SBP	LNC		SBP	LNC		SBP	LNC	
	Mean ± SE		*P*-value*	Mean ± SE		*P*-value*	Mean ± SE		*P*-value*
pH	6.2 ± 0.1	6.1 ± 0.1	0.360	6.0 ± 0.1	6.2 ± 0.2	0.776	6.5 ± 0.1	6.7 ± 0.2	1.000
Acetate	84.3 ± 5.7	81.0 ± 5.3	0.739	93.2 ± 7.4	75.5 ± 2.6	0.113	93.2 ± 4.1	74.6 ± 6.9	0.053
Propionate	33.7 ± 2.7	34.0 ± 1.8	0.853	44.9 ± 4.5	35.8 ± 2.5	0.033	39.6 ± 2.9	30.0 ± 3.5	0.053
i-Butyrate	3.8 ± 0.4	3.1 ± 0.2	0.133	3.3 ± 0.5	2.4 ± 0.2	< 0.001	4.7 ± 0.4	3.3 ± 0.3	0.013
n-Butyrate	21.2 ± 1.9	21.8 ± 2.0	0.971	27.9 ± 2.2	26.0 ± 1.9	0.475	22.1 ± 1.9	18.1 ± 2.6	0.243
i-Valerate	5.1 ± 0.7	3.9 ± 0.3	0.190	5.4 ± 0.8	3.4 ± 0.4	0.004	7.0 ± 0.6	5.0 ± 0.5	0.043
n-Valerate	3.4 ± 0.4	3.6 ± 0.5	0.971	4.4 ± 0.5	3.8 ± 0.3	0.796	4.9 ± 0.5	3.9 ± 0.4	0.408
Total SCFA	151.5 ± 11.0	147.5 ± 8.2	1.000	179.2 ± 12.6	147.0 ± 6.4	0.007	171.5 ± 8.9	135.0 ± 13.3	0.014
l-Lactate	0.53 ± 0.09	1.04 ± 0.41	1.000	0.96 ± 0.37	0.79 ± 0.23	0.829	0.22 ± 0.04	0.40 ± 0.06	0.083
d-Lactate	0.18 ± 0.04	0.60 ± 0.25	0.661	0.71 ± 0.25	0.59 ± 0.13	0.963	0.13 ± 0.04	0.27 ± 0.06	0.234
Ammonia	32.9 ± 4.5	23.5 ± 1.9	0.237	30.0 ± 4.7	18.7 ± 1.8	0.043	29.9 ± 2.1	20.9 ± 2.1	0.008
d/l-lactate ratio	0.32 ± 0.04	0.66 ± 0.13	0.043	0.76 ± 0.09	2.0 ± 1.03	0.815	0.62 ± 0.15	0.53 ± 0.07	1.000
Total microbial metabolites**	185.1 ± 14.9	172.7 ± 9.6	0.739	210.8 ± 13.5	167.1 ± 6.9	0.003	203.7 ± 11.3	156.5 ± 14.8	0.011
Putrescine	0.16 ± 0.06	0.11 ± 0.02	0.122	0.49 ± 0.19	0.13 ± 0.04	0.400	0.04 ± 0.01	0.03 ± 0.01	0.340
Histamine	0.01 ± 0	0.01 ± 0.002	–	0.03 ± 0.02	0.01 ± 0.002	0.517	0	0	-
Cadaverine	0.04 ± 0.01	0.08 ± 0.02	0.190	0.47 ± 0.26	0.17 ± 0.06	0.028	0.03 ± 0.01	0.05 ± 0.02	0.965
Spermidine	0.28 ± 0.03	0.19 ± 0.01	< 0.001	0.29 ± 0.10	0.17 ± 0.02	0.505	0.13 ± 0.01	0.08 ± 0.01	0.008
Tyramine	0.001 ± 0	0.03 ± 0	–	0.09 ± 0.08	0	–	0	0	-
Spermine	0.01 ± 0.001	0.01 ± 0.001	0.315	0.01 ± 0.0001	0.01 ± 0.002	0.001	0.01 ± 0	0.01 ± 0.002	-
Total biogenic amines	0.49 ± 0.09	0.40 ± 0.04	0.815	1.30 ± 0.55	0.48 ± 0.10	0.200	0.20 ± 0.02	0.17 ± 0.03	0.278

*SCFA* short chain fatty acids

*Total microbial metabolites: sum of SCFA, l-Lactate, d-Lactate and ammonia

### Piglet Fecal Score

**Fig. 2:**
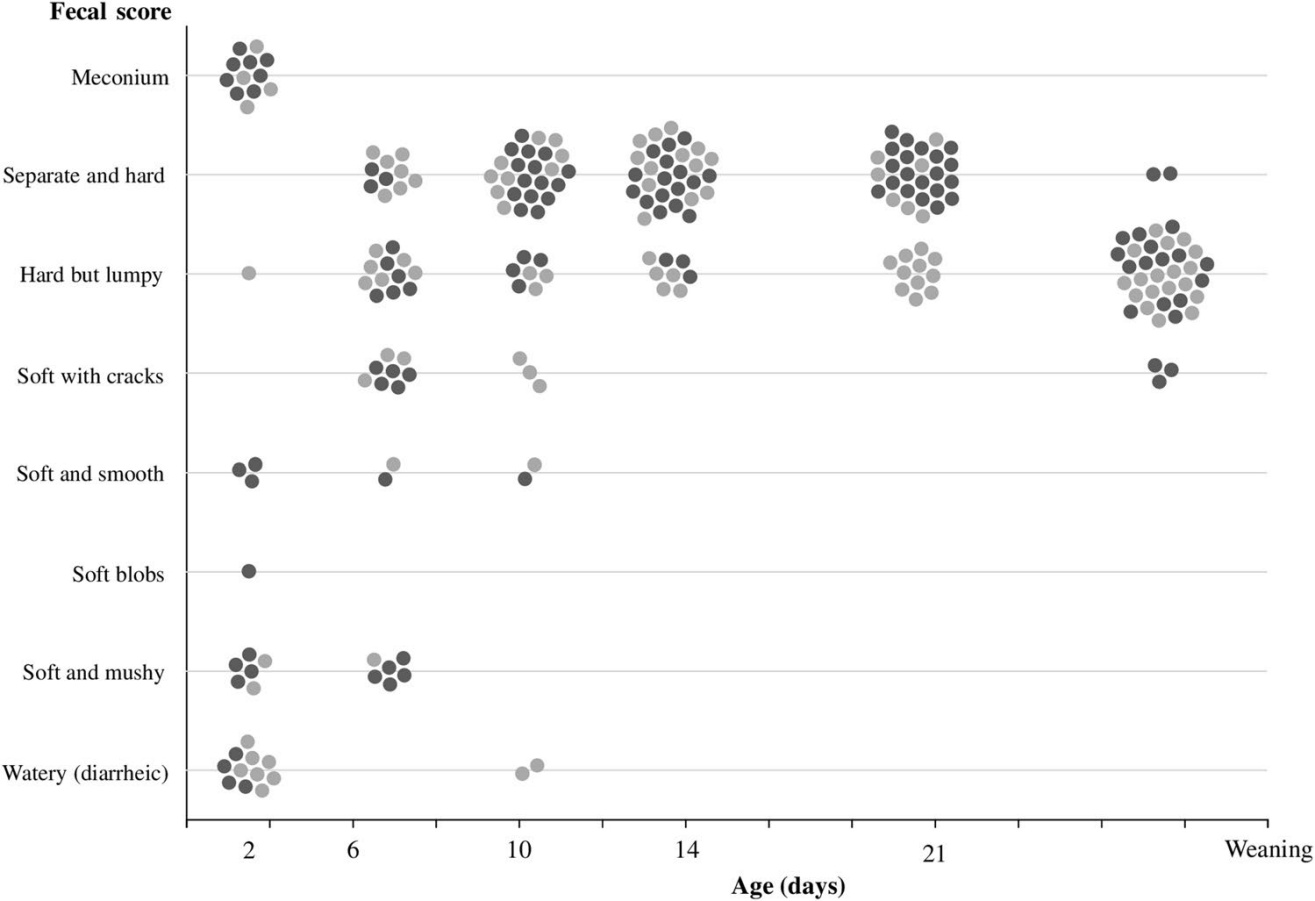
Fecal score in piglet feces whose mother sows were fed diets containing high-fermentable sugar beet pulp (light-grey points) or low-fermentable lignocellulose (dark-grey points) fibers during gestation and lactation. Each dot represents an individual piglet faecal sample. The seven-scale “Bristol stool form scale” was adapted to assess the fecal score for all sow and all piglet samples, in which an additional score (“0”) was included if meconium was present in piglet feces. The fecal score was as follows: 0, meconium; 1, separate and hard; 2, hard but lumpy; 3, soft with cracks; 4, soft and smooth; 5, soft blobs; 6, soft and mushy; 7, watery (diarrheic)

The fecal score of the study piglets is shown in Fig. 2. Two days after birth, majority of piglets from SBP and LNC groups defecated meconium or soft to watery feces. At six and 10 days of life, majority of feces had a hard consistency in both groups. Fourteen- and 21-day-old piglets from the LNC group presented more constipated stools than piglets from the SBP group. At weaning, piglets from SBP group had softer feces than from the LNC group.

### Colonization of Piglets by *C. difficile*

**Fig. 3:**
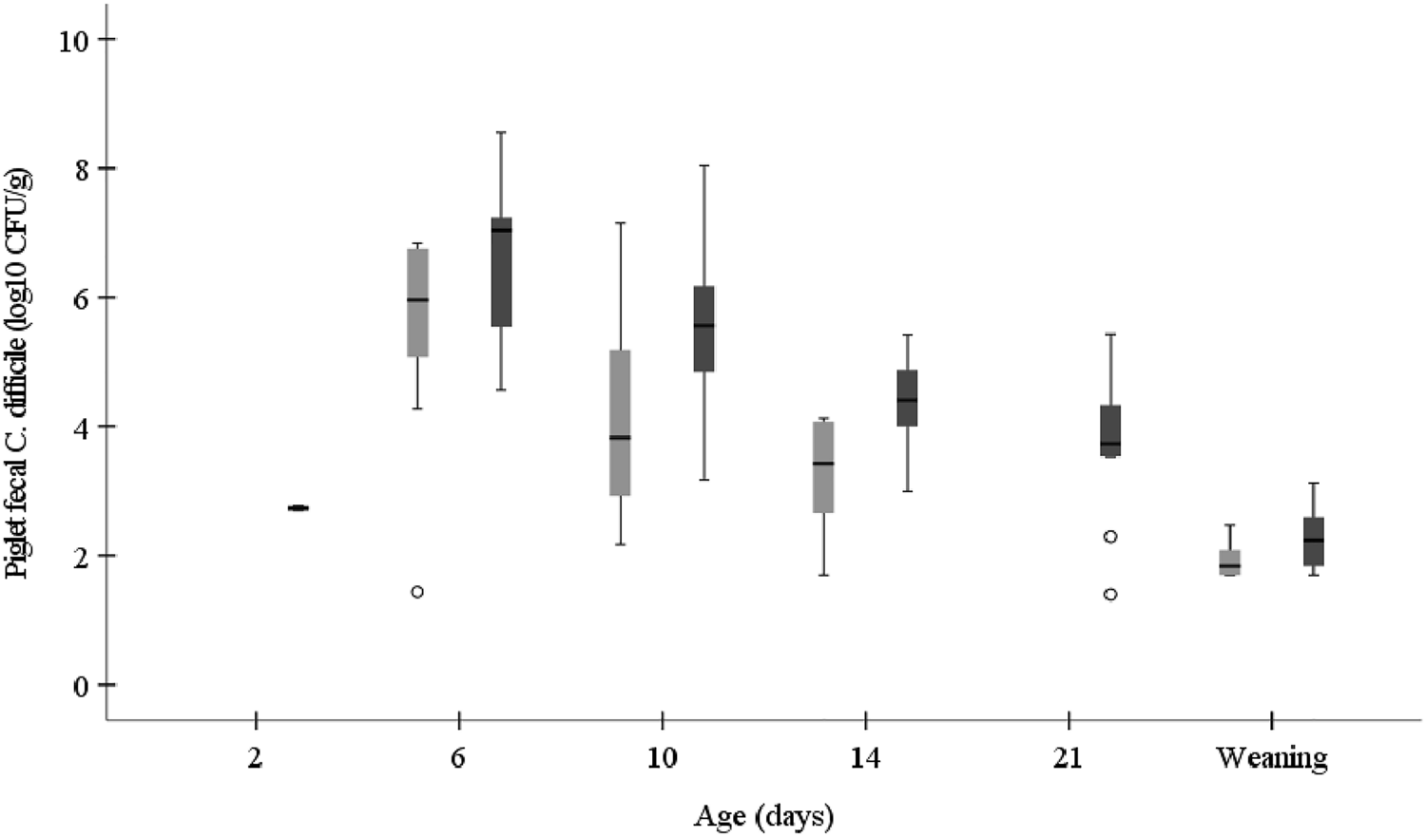
Concentration of *C. difficile* (log_10_ CFU/g) in piglet feces whose mother sows were fed diets containing high-fermentable sugar beet pulp (SBP) or low-fermentable lignocellulose (LNC) fibers during gestation and lactation. Circles indicate outliers. Sample number (SBP/LNC) in each boxplot: 2d: 0/2; 6d: 13/13; 10d: 15/16; 14d: 8/13; 21d: 0/9; weaning: 8/12

*C. difficile* was already detected in two-day-old piglets from LNC group (log_10_ 2.7 CFU/g) but not in piglets from SBP group. Concentrations of *C. difficile* were significantly lower in six-, 10- and 14-day-old piglets from the sows fed SBP than LNC diets (log_10_ 6.3 CFU/g vs. log_10_ 7.0 CFU/g, *P* = 0.034; log_10_ 3.8 CFU/g vs. log_10_ 5.6 CFU/g, *P* = 0.024; log_10_ 3.4 CFU/g *vs*. log_10_ 4.4 CFU/g, *P* = 0.010, respectively). In three-week-old piglets from the LNC group *C. difficile* concentration was log_10_ 3.7 CFU/g, while the bacterium was not detected in piglets from SBP group. At weaning, concentrations of *C. difficile* were lower in piglets from SBP as compared to LNC group (log_10_ 1.9 CFU/g *vs*. log_10_ 2.2 CFU/g, *P* = 0.098) (Fig. 3).

The percentage of piglets shedding *C. difficile* tended to be higher in piglets from the sows fed LNC than SBP diets along the trial (Table 3). At day 21, significantly lower percentage of piglets from the sows fed SBP than LNC shed *C. difficile* in their feces (0% vs. 45.0%, *P* = 0.001).

**Table 3:** Prevalence (percentage of positive samples) of *C. difficile* in piglet feces whose mother sows were fed diets containing high-fermentable sugar beet pulp (SBP) or low-fermentable lignocellulose (LNC) fibers during gestation and lactation

Day	SBP	LNC	
	% prevalence (positive/total)		*P*-value
2	0 (0/13)	10.5 (2/19)	0.502
6	86.7 (13/15)	100.0 (13/13)	0.484
10	83.3 (15/18)	84.2 (16/19)	1.000
14	44.4 (8/18)	65.0 (13/20)	0.328
21	0 (0/18)	45.0 (9/20)	0.001
Weaning	44.4 (8/18)	60.0 (12/20)	0.516

### Toxin B in Piglets

**Fig. 4:**
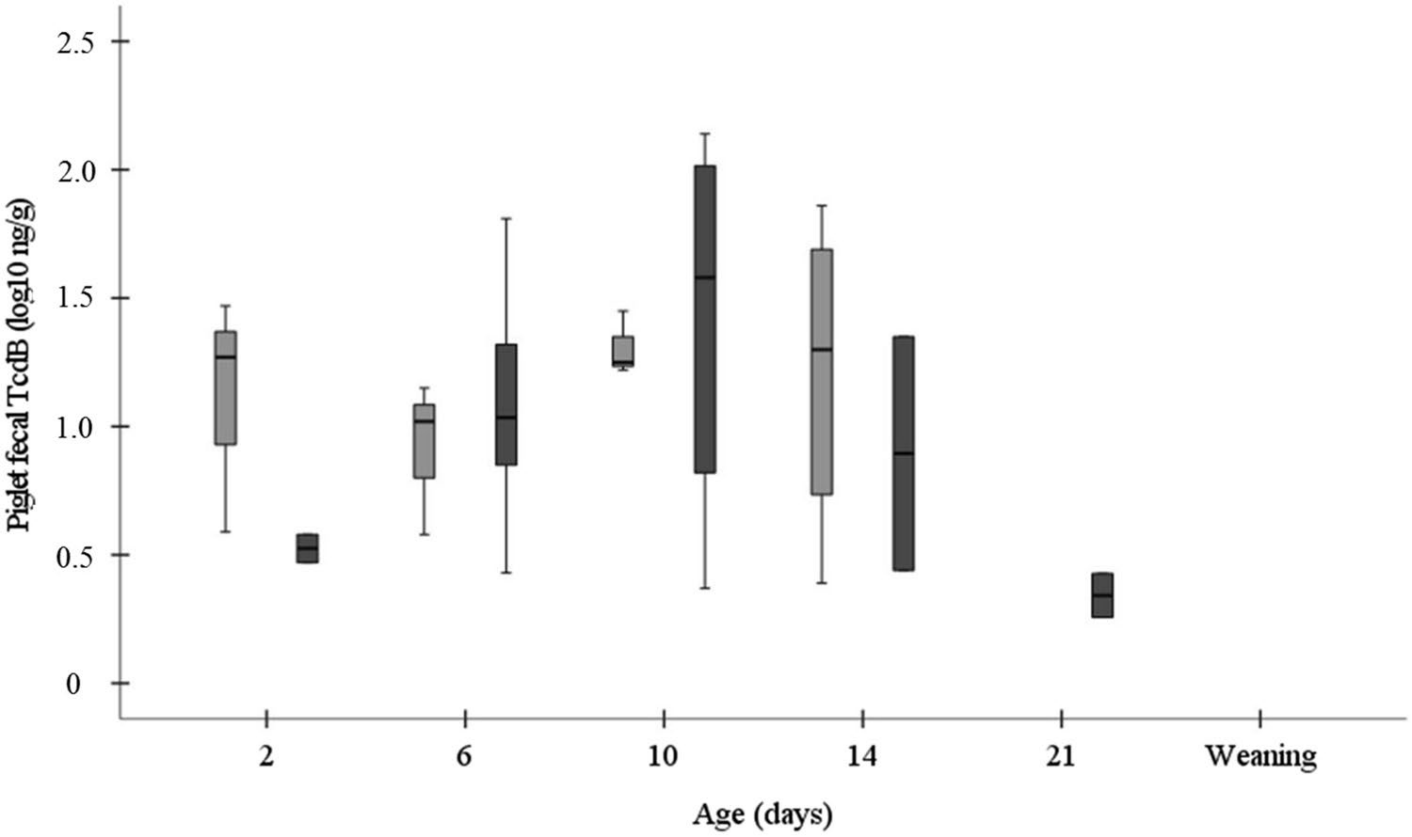
Concentration of TcdB (log_10_ ng/g) in piglet feces whose mother sows were fed diets containing high-fermentable sugar beet pulp (SBP) or low-fermentable lignocellulose (LNC) fibers during gestation and lactation. Sample number (SBP/LNC) in each boxplot: 2d: 3/2; 6d: 3/12; 10d: 3/4; 14d: 4/2; 21d: 0/2; weaning: 0/0

TcdB was detected already in feces from two-day-old piglets, however at a low concentration in both feeding groups (log_10_ 1.3 ng/g vs. log_10_ 0.5 ng/g, *P* = 0.200). The concentration of TcdB in six-day-old piglets was log_10_ 1.0 ng/g and log_10_ 1.0 ng/g in piglets from SBP and LNC group (*P* = 0.840), respectively. At 10 days of age TcdB was lower in piglets from SBP, as compared to LNC group (log_10_ 1.3 ng/g vs. log_10_ 1.6 ng/g, *P* = 0.629). Fourteen-day-old piglets from SBP and LNC groups carried log_10_ 1.3 ng/g and log_10_ 0.9 ng/g of TcdB (*P* = 0.800), respectively. At 3 weeks of age the TcdB was not detected in piglets from SBP group, but it was determined at a concentration of log_10_ 0.3 ng/g in piglets from LNC group (Fig. 4).

At six days of age, significantly more piglets from the LNC group were positive for TcdB, as compared to piglets from the SBP group (60.0% vs. 16.7%, *P* = 0.009) (Table 4).

**Table 4:** Prevalence (percentage of positive samples) of TcdB in piglet feces whose mother sows were fed diets containing high-fermentable sugar beet pulp (SBP) or low-fermentable lignocellulose (LNC) fibers during gestation and lactation

Day	SBP	LNC	
	% prevalence (positive/total)		*P*-value
2	16.7 (3/18)	10.0 (2/20)	0.653
6	16.7 (3/18)	60.0 (12/20)	0.009
10	16.7 (3/18)	20.0 (4/20)	1.000
14	22.2 (4/18)	10.5 (2/19)	0.405
21	0 (0/18)	10.0 (2/20)	0.488
Weaning	0 (0/18)	0 (0/19)	–

Concentration of *C. difficile* was positively correlated with the concentration of TcdB (*r* = 0.568, *n* = 23, *P* = 0.005).

### Bacterial Communities and Diversity Indices in Piglet Feces

In the individual piglet fecal samples, we found between 12 and 113 bacterial taxa. The total number of identified bacterial taxa was 172, of which the 26 dominant taxa were displayed in stacked bar plots. (Supplementary Figure S2). Here, taxa of *Lactobacillus* spp., *Clostridium* sensu stricto 1 and sequences belonging to *Muribaculaceae* family predominated the gut microbiota of piglets. A significantly higher abundance of *Lachnoclostridium* spp. (*P* = 0.029) and *Bifidobacterium* spp. (*P* = 0.029) were found in the feces of 14-day-old piglets from sows fed LNC vs. SBP. Here, there was trend for a higher abundance of *Prevotella* spp. (*P* = 0.057) in the piglets from sows fed SBP vs. LNC. One week later, taxa of *Coprococcus* 3 spp. (*P* = 0.029) and *Terrisporobacter* spp. (*P* = 0.029) predominated in the feces of piglets from sows fed SBP vs. LNC while the abundance of *Escherichia*-*Schigella* taxon (*P* = 0.029) was significantly higher in piglets from sows fed LNC vs. SBP. At weaning, a significantly higher abundance of *Terrisporobacter* spp. (*P* = 0.029) and a trend for a higher abundance of *Coprococcus* 3 spp. (*P* = 0.057) taxa were found in piglets from sows fed SBP vs. LNC. On the contrary, a significantly higher abundance of *Ruminococcus* spp. (*P* = 0.029) was detected in piglets from sows fed LNC vs. SBP.

**Fig. 5:**
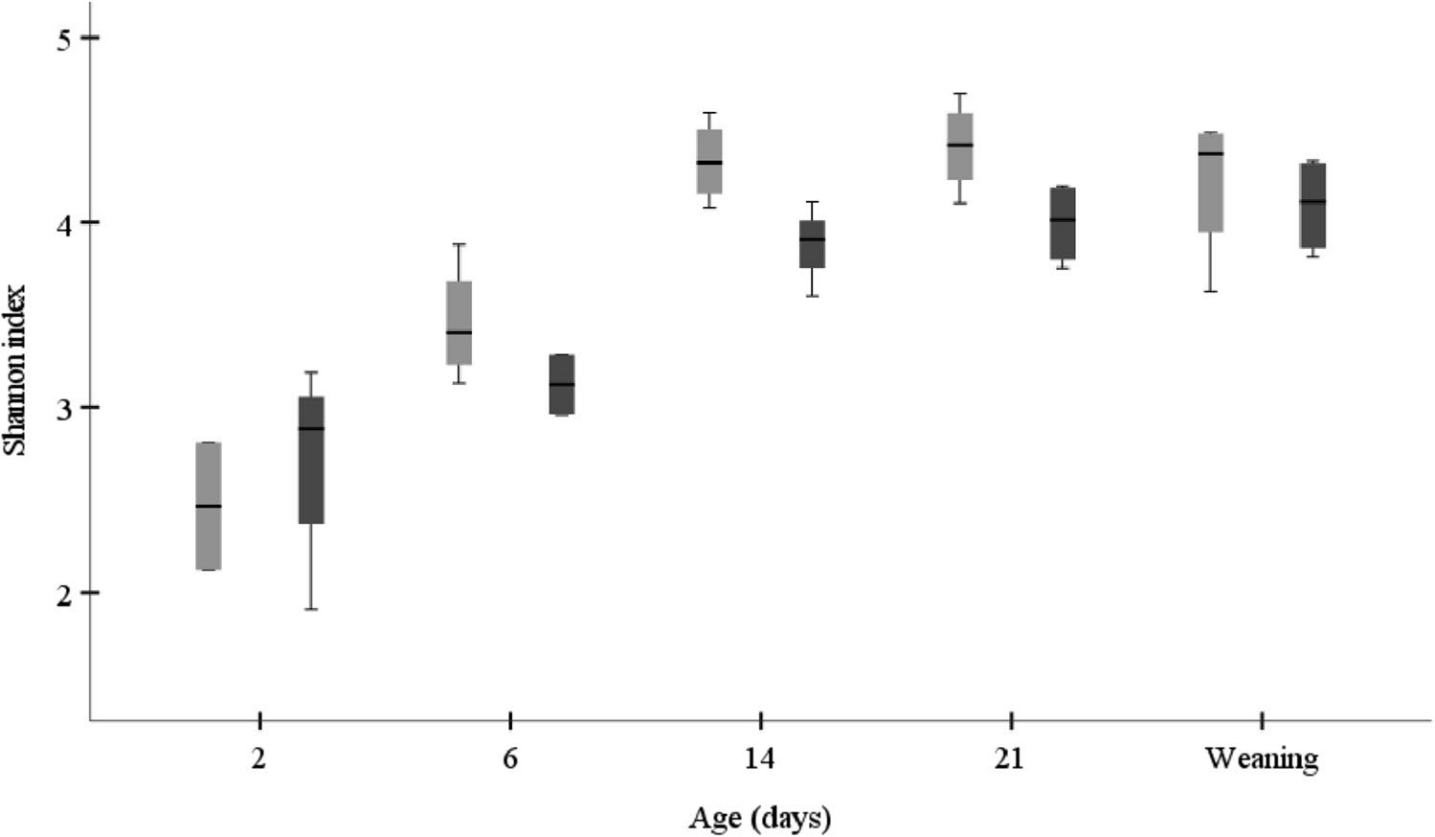
Diversity shown as Shannon index using the relative abundance of ASV in piglet feces (*n* = 4/age/group) whose mother sows (*n* = 4/age/group) were fed diets containing high-fermentable sugar beet pulp (SBP) or low-fermentable lignocellulose (LNC) fibers during gestation and lactation, as analyzed by the 16S-rDNA sequencing. Light-grey bars represent SBP, while dark-grey bars represent LNC

In piglets, microbial diversity represented by Shannon index gradually increased as the animals aged (Fig. 5). At 2 weeks of age, there was a trend for a higher bacterial Shannon diversity index in piglets from sows fed SBP vs. LNC (*P* = 0.057).

## Discussion

The influence of the mother on the offspring ontogeny and health by different dietary means in animals and humans has gained increasing interest. It has been demonstrated that the association between mother and infant is important for the maturation of the gut microbiota and immune system since events taking place during perinatal period have important implications in early life and adulthood [19, 39]. However, little is known about the mother–offspring association in pigs, especially considering microbial imprinting during the neonatal period. Moreover, sow-originated factors may contribute to a protection against microbial pathogens including *C. difficile* in piglets. Indeed, sow nutrition during pregnancy and lactation has been shown to influence the health and wellbeing of their piglets [17, 40].

Here, we assessed the effect of sows’ diet enriched in highly- or low-fermentable fiber sources on the colonization of piglets by *C. difficile*. In this study, the sows were fed diets containing higher concentrations of either high-fermentable sugar beet pulp or low-fermentable lignocellulose during gestation and lactation periods. Our results show that those suckling piglets whose mothers consumed diets enriched with sugar beet pulp had lower concentrations of *C. difficile* in their feces compared to piglets whose mothers consumed diets enriched with lignocellulose. Suckling piglets of the sows fed sugar beet pulp were also less likely to be colonized by *C. difficile* along the suckling period, as compared to their counterparts. Lower concentrations of toxin B were detected in piglets from sows fed sugar beet pulp, as compared to lignocellulose. In addition, the prevalence of piglets in which toxin B was detected was lower in one-week-old animals from sows fed sugar beet pulp compared to lignocellulose.

*C. difficile* colonizes the piglets gut at birth and its concentration increases rapidly during the first week of life [14]. Among *C. difficile*, virulent types are present which are able to produce toxins such as toxin A and/or B, leading to gut intoxication and CDI progress [41]. In our study, the microbial diversity as assessed by Shannon index, increased with the piglets’ age. The developing and yet immature gut microbiota characterized by a low diversity, as we observed in the present and earlier studies, may open a niche for *C. difficile* successful colonization in suckling piglets [2]. However, due to different fecal sample number for *C. difficile* and microbiota determination, the analysis of a direct effect of microbiota on *C. difficile* could not be performed. Further, any disruption of the natural colonization process or perturbances of the intestinal ecosystem could increase the chances of piglets developing CDI [41]. In a dynamic and complex gut ecosystem, certain bacterial groups may be in intimate contact with each other, entering different ecological dependences. Likewise, infants treated with antimicrobials or suffering from bacterial or viral gut infections are at higher risk of developing CDI [42]. In suckling piglets, CDI is characterized by diarrhea or constipation, increased body temperature and a presence of *C. difficile* and its toxins in feces [13]. In this study, we quantified toxin B only, since more CDI outbreaks are associated with *C. difficile* producing either both toxins or toxin B, rather than toxin A only [43].

A limited number of studies assessed the impact of dietary intervention on *C. difficile* colonization in animals. For instance, atherogenic, axenic or elemental diets fed to hamsters and mice caused an increased *C. difficile* proliferation and toxin synthesis in the gut, which resulted in CDI development and lower survival rates of the animals [44–47]. In humans, previous reports demonstrated increased levels and prevalence of *C. difficile* in formula fed *vs.* breastfed infants [5]. Moreover, *C. difficile* was more often detected in infants from high-income *vs.* low-income countries [48], suggesting that a maternal diet, often high in processed foods in high-income countries and high in fiber in low-income countries, can be directly or indirectly related to *C. difficile* colonization in infants. In our study, the results clearly suggest a protective impact of the addition of high fermentable fibers over low fermentable fibers in sow’s feeds during gestation and lactation periods against the colonization of piglets by *C. difficile*. Specifically, a more diverse microbiota was found during periparturient period in sows fed sugar beet pulp, as compared to sows fed lignocellulose. Following this finding, suckling piglets from the sows fed sugar beet pulp compared to lignocellulose tended to have a higher microbial diversity, supporting the hypothesis of the mother–offspring microbial programming and microbial diversity being associated with *C. difficile* colonization [2, 8]. Similarly, a previous study showed that highly fermentable non-starch-polysaccharides, such as inulin added to the diet can modulate certain bacterial groups (as analyzed by qPCR) of gestating and lactating sows and their suckling piglets [49]. Inclusion of certain carbohydrates which are not digestible for pigs but for gut microorganisms is known to influence the microbial metabolic activity [37]. Here, metabolites were assessed in sow feces collected during the periparturient period i.e. one week ante-partum, at the farrowing and one week post-partum, as in this period the sow feces have a direct contact with a newborn piglet which may potentially influence the early microbial programming in the offspring. We found that the addition of sugar beet pulp to the sows’ diet during gestation and lactation increased production of SCFA, certain biogenic amines and ammonia in the feces during the periparturient period, as compared to sows fed diets enriched with lignocellulose. Sugar beet pulp has been shown to be more easily degradable by the gut microbiota, due to its higher solubility and accessibility to microbial enzymes than lignocellulose, resulting in a production of higher levels of microbial metabolites [50]. Previous in vitro studies have demonstrated a negative impact of high levels of SCFA and low pH on *C. difficile* growth and toxin production [51, 52]. Clinical data demonstrate that butyrate-producing anaerobic bacteria were significantly depleted in the CDI patients [6]. Recently, stool samples from patients with CDI had lower valerate concentrations, which were restored after fecal microbial transplantation and additionally, in chemostat models valerate decreased vegetative growth of *C. difficile* [53]. Although an increased microbial metabolic activity in the sows fed sugar beet pulp did not influence *C. difficile* shedding by the sows compared to sows fed lignocellulose, a higher concentration of metabolites in the sows’ feces may have influenced *C. difficile* colonization in their piglets. Pigs are known for coprophagy and nursing piglets have a constant contact with sow’s feces after birth which may facilitate the influence of sows fecal microecology on their offspring [54]. Considering the yet immature gut microecosystem in neonatal piglets and its vulnerability to environmental factors, changes in microbial composition accompanied by higher concentrations of SCFA in the feces of sows fed sugar beet pulp may have reduced *C. difficile* proliferation in suckling piglets. To understand these specific relationships, targeted in vitro and ex vivo approaches may be necessary.

Farrowing is a stressful period for mammals, which has a negative impact on physiology, immune system and microbial diversity [55, 56]. Indeed, we have previously observed shifts in the gut microbiota composition in sows during periparturient period and such changes may possibly lead to an increased susceptibility to certain infections [2]. Similarly, microbial dysbiosis accompanied by an increase in proinflammatory cytokines and energy loss has been observed in healthy pregnant women [55, 57]. Thus, specific dietary strategies could offer an opportunity to influence the gut ecology and health of the host. It is noteworthy that one week after farrowing, all sows had detectable levels of *C. difficile* in their feces. Interestingly, since *C. difficile* rapidly colonizes one-week-old neonatal piglets, it is very likely that a high load of *C. difficile* excreted with the piglet’s feces re-inoculated the sows due to constant sow-piglet contact and coprophagy during the nursing period [54]. The proposed “re-inoculation” phenomenon of *C. difficile* to the sows, could possibly possess a risk of CDI, if in addition the sows were treated with antimicrobials which are a contributing factor to CDI development in young pigs or in humans, as reviewed previously [8]. If the “re-inoculation” phenomenon were true, it would mean that not only a sow could influence the microbial ecosystem of their piglets but also vice versa. Such observation may have important consequences for the health of the sows during periparturient period and should be investigated in future studies. Likewise, high prevalence of toxigenic *C. difficile* observed in infant feces could contribute to pathogen dissemination in a community and possess a risk of infection in adults suffering from gut microbial dysbiosis [58]. This can have important public health implications and should be studied in more detail.

The awareness of the prenatal and postnatal environment is fundamental for the early microbial and immune programming. Mammal offspring are in the intimate contact with their mothers already in utero following the nursing period [16]. In humans, it is known that the maternal microbiota, immune system and metabolism have a profound impact on infant development [59]. In intensive production systems, neonatal piglets are more vulnerable to stress and gut dysbiosis [60]. Identifying the beneficial effects of dietary components and their roles during gestation and lactation may have promising implications in microbiota programming of the offspring, controlling of pathogen colonization and dissemination, and reducing the risk of diseases. Finally, the opportunity to improve piglet health through the sow-offspring association by dietary means offers an attractive approach to control piglet’s resilience to gut pathogens, such as *C. difficile*.

Taken together, sow’s diets enriched with highly fermentable sugar beet pulp, compared to low fermentable lignocellulose dietary fibers during gestation and lactation reduced *C. difficile* shedding in suckling piglets. Susceptibility to colonization by gut pathogens, such as *C. difficile* in neonatal piglets can be influenced by the sows’ nutritional factors supporting the phenomenon of the mother–offspring early microbial programming.

## Supplementary Information

Below is the link to the electronic supplementary material.

Supplementary file1 (PDF 49 kb)

Supplementary file2 (PDF 48 kb)

## Abbreviations

*C. difficile*: *Clostridioides difficile*
CDI: *Clostridioides difficile* Infection
TcdB: Toxin B
SBP: Sugar beet pulp
LNC: Lignocellulose
ELISA: Enzyme-linked immunosorbent assay
ASV: Amplicon sequence variants

## References

[1] Bian G, Ma S, Zhu Z (2016). Age, introduction of solid feed and weaning are more important determinants of gut bacterial succession in piglets than breed and nursing mother as revealed by a reciprocal cross-fostering model. Environ Microbiol.

[2] Grześkowiak D, Zentek V (2019). Developing gut microbiota exerts colonisation resistance to clostridium (syn. Clostridioides) difficile in piglets. Microorganisms.

[3] Li Y, Liu H, Zhang L (2020). Maternal dietary fiber composition during gestation induces changes in offspring antioxidative capacity, inflammatory response, and gut microbiota in a sow model. Int J Mol Sci.

[4] Theil PK, Lauridsen C, Quesnel H (2014). Neonatal piglet survival: impact of sow nutrition around parturition on fetal glycogen deposition and production and composition of colostrum and transient milk. Animal.

[5] Grześkowiak Ł, Grönlund M-M, Beckmann C (2012). The impact of perinatal probiotic intervention on gut microbiota: double-blind placebo-controlled trials in Finland and Germany. Anaerobe.

[6] Antharam VC, Li EC, Ishmael A (2013). Intestinal dysbiosis and depletion of butyrogenic bacteria in *Clostridium difficile* infection and nosocomial diarrhea. J Clin Microbiol.

[7] Sun J, Du L, Li XL (2019). Identification of the core bacteria in rectums of diarrheic and non-diarrheic piglets. Sci Rep.

[8] Grześkowiak ŁM, Pieper R, Huynh HA (2019). Impact of early-life events on the susceptibility to *Clostridium difficile* colonisation and infection in the offspring of the pig. Gut Microbes.

[9] Grönlund M, Grzeskowiak Ł, Isolauri E, Salminen S (2011). Influence of mother’s intestinal microbiota on gut colonization in the infant. Gut Microbes.

[10] Gueimonde M, Sakata S, Kalliomäki M (2006). Effect of maternal consumption of lactobacillus GG on transfer and establishment of fecal bifidobacterial microbiota in neonates. J Pediatr Gastroenterol Nutr.

[11] Friedemann M (2009). Epidemiology of invasive neonatal Cronobacter (*Enterobacter sakazakii*) infections. Eur J Clin Microbiol Infect Dis.

[12] Grześkowiak Ł, Martínez-Vallespín B, Dadi TH (2018). Formula feeding predisposes neonatal piglets to *Clostridium difficile* gut infection. J Infect Dis.

[13] Songer JG, Anderson MA (2006). *Clostridium difficile*: an important pathogen of food animals. Anaerobe.

[14] Grześkowiak Ł, Zentek J, Vahjen W (2016). Determination of the extent of *Clostridium difficile* colonisation and toxin accumulation in sows and neonatal piglets. Anaerobe.

[15] Rolfe RD, Song W (1995). Immunoglobulin and non-immunoglobulin components of human milk inhibit *Clostridium difficile* toxin A-receptor binding. J Med Microbiol.

[16] Everaert N, Van Cruchten S, Weström B (2017). A review on early gut maturation and colonization in pigs, including biological and dietary factors affecting gut homeostasis. Anim Feed Sci Technol.

[17] Shang Q, Liu H, Liu S (2019). Effects of dietary fiber sources during late gestation and lactation on sow performance, milk quality, and intestinal health in piglets. J Anim Sci.

[18] Loisel F, Farmer C, Ramaekers P, Quesnel H (2013). Effects of high fiber intake during late pregnancy on sow physiology, colostrum production, and piglet performance. J Anim Sci.

[19] Kalliomäki M, Salminen S, Arvilommi H (2001). Probiotics in primary prevention of atopic disease: a randomised placebo-controlled trial. Lancet.

[20] Collado MC, Isolauri E, Laitinen K, Salminen S (2010). Effect of mother ’s weight on infant ’s microbiota acquisition, composition, and activity during early infancy : a prospective follow-up study initiated in early pregnancy. Am J Clin Nutr.

[21] Krogh U, Bruun TS, Amdi C (2015). Colostrum production in sows fed different sources of fiber and fat during late gestation. Can J Anim Sci.

[22] Li Y, Zhang L, Liu H (2019). Effects of the ratio of insoluble fiber to soluble fiber in gestation diets on sow performance and offspring intestinal development. Animals.

[23] GfE, (2006). Empfehlungen zur Energie- und Nährstoffversorgung von Schweinen.

[24] Naumann C, Bassler R, Seibold R, Barth C (1976) Methodenbuch. Band III, Band III. VDLUFA - Verlag, Darmstadt

[25] Lewis SJ, Heaton KW (1997). Stool form scale as a useful guide to intestinal transit time. Scand J Gastroenterol.

[26] Grześkowiak Ł, Zentek J, Vahjen W (2016). Physical pre-treatment improves efficient DNA extraction and qPCR sensitivity from *Clostridium difficile* spores in faecal swine specimens. Curr Microbiol.

[27] Bushnell B, Rood J, Singer E (2017). BBMerge: accurate paired shotgun read merging via overlap. PLoS ONE.

[28] Bolyen E, Rideout JR, Dillon MR (2018). QIIME 2: reproducible, interactive, scalable, and extensible microbiome data science. PeerJ Prepr.

[29] Callahan BJ, McMurdie PJ, Rosen MJ (2016). DADA2: high-resolution sample inference from Illumina amplicon data. Nat Methods.

[30] Callahan BJ, McMurdie PJ, Holmes SP (2017). Exact sequence variants should replace operational taxonomic units in marker-gene data analysis. ISME J.

[31] Weiss S, Xu ZZ, Peddada S (2017). Normalization and microbial differential abundance strategies depend upon data characteristics. Microbiome.

[32] Bokulich NA, Kaehler BD, Rideout JR (2018). Optimizing taxonomic classification of marker-gene amplicon sequences with QIIME 2’s q2-feature-classifier plugin. Microbiome.

[33] Pedregosa F, Varoquaux G, Gramfort A (2011). Scikit-learn: machine learning in Python. J Mach Learn Res.

[34] Yilmaz P, Parfrey LW, Yarza P (2014). The SILVA and “all-species Living Tree Project (LTP)” taxonomic frameworks. Nucleic Acids Res.

[35] Oksanen AJ, Blanchet FG, Friendly M (2019). Vegan. Encycl Food Agric Ethics.

[36] Spellerberg IF, Fedor PJ (2003). A tribute to Claude-Shannon (1916–2001) and a plea for more rigorous use of species richness, species diversity and the “Shannon-Wiener” Index. Glob Ecol Biogeogr.

[37] Pieper R, Boudry C, Bindelle J (2014). Interaction between dietary protein content and the source of carbohydrates along the gastrointestinal tract of weaned piglets. Arch Anim Nutr.

[38] Mauri M, Elli T, Caviglia G, et al (2017) RAWGraphs: a visualisation platform to create open outputs. In: Proceedings of the 12th biannual conference on Italian SIGCHI Chapter. Association for Computing Machinery, New York, NY, USA

[39] Marko K, Seppo S, Tuija P, Arvilommi Heikki IE (2003). Probiotics and prevention of atopic disease : 4-year follow-up of a randomised placebo-controlled trial For personal use. Only reproduce with permission from The Lancet Publishing Group. Lancet.

[40] Quesnel H, Meunier-Salaün MC, Hamard A (2009). Dietary fiber for pregnant sows: influence on sow physiology and performance during lactation. J Anim Sci.

[41] Grzeskowiak L, Martinez-Vallespin B, Dadi TH (2017). Formula-feeding predisposes neonatal piglets to *Clostridium difficile* gut infection. J Infect Dis.

[42] Lees EA, Miyajima F, Pirmohamed M, Carrol ED (2016). The role of *Clostridium difficile* in the paediatric and neonatal gut: a narrative review. Eur J Clin Microbiol Infect Dis.

[43] Drudy D, Fanning S, Kyne L (2007). Toxin A-negative, toxin B-positive *Clostridium difficile*. Int J Infect Dis.

[44] Mahe S, Corthier G, Dubos F (1987). Effect of various diets on toxin production by two strains of *Clostridium difficile* in gnotobiotic mice. Infect Immun.

[45] Frankel WL, Choi DM, Zhang W (1994). Soy fiber delays disease onset and prolongs survival in experimental *Clostridium difficile* ileocecitis. JPEN J Parenter Enteral Nutr.

[46] Blankenship-Paris TL, Walton BJ, Hayes YO, Chang J (1995). *Clostridium difficile* infection in hamsters fed an atherogenic diet. Vet Pathol.

[47] Iizuka M, Itou H, Konno S (2004). Elemental diet modulates the growth of *Clostridium difficile* in the gut flora. Aliment Pharmacol Ther.

[48] Grześkowiak Ł, Collado MC, Mangani C (2012). Distinct gut microbiota in Southeastern African and Northern European infants. J Pediatr Gastroenterol Nutr.

[49] Paßlack N, Vahjen W, Zentek J (2015). Dietary inulin affects the intestinal microbiota in sows and their suckling piglets. BMC Vet Res.

[50] Pieper R, Kroger S, Richter JF (2012). Fermentable fiber ameliorates fermentable protein-induced changes in microbial ecology, but not the mucosal response, in the colon of piglets. J Nutr.

[51] Karlsson S, Lindberg A, Norin E (2000). Toxins, butyric acid, and other short-chain fatty acids are coordinately expressed and down-regulated by cysteine in *Clostridium difficile*. Infect Immun.

[52] May T, Mackie RI, Fahey GC (1994). Effect of fiber source on short-chain fatty acid production and on the growth and toxin production by *Clostridium difficile*. Scand J Gastroenterol.

[53] McDonald JAK, Mullish BH, Pechlivanis A (2018). Inhibiting growth of *Clostridioides difficile* by restoring valerate, produced by the intestinal microbiota. Gastroenterology.

[54] Whatson TS, Bertram JM (1983). Some observations on mother-infant interactions in the pig (Sus scrofa). Appl Anim Ethol.

[55] Koren O, Goodrich JK, Cullender TC (2012). Host remodeling of the gut microbiome and metabolic changes during pregnancy. Cell.

[56] Nuriel-Ohayon M, Neuman H, Koren O (2016). Microbial changes during pregnancy, birth, and infancy. Front Microbiol.

[57] Collado MC, Isolauri E, Laitinen K, Salminen S (2008). Distinct composition of gut microbiota during pregnancy in overweight and normal-weight women. Am J Clin Nutr.

[58] Smits WK, Lyras D, Lacy DB (2016). Clostridium difficile infection. Nat Rev Dis Prim.

[59] Milani C, Duranti S, Bottacini F (2017). The first microbial colonizers of the human gut: composition, activities, and health implications of the infant gut microbiota. Microbiol Mol Biol Rev.

[60] Pluske JR, Turpin DL, Kim JC (2018). Gastrointestinal tract (gut) health in the young pig. Anim Nutr.

[61] Knudsen KEB (1997). Carbohydrate and lignin contents of plant materials used in animal feeding. Anim Feed Sci Technol.

