# Supplementary figures and images for: Fiber Composition in Sows’ Diets Modifies Clostridioides difficile Colonization in Their Offspring
Source: Curr Microbiol. 2022 Apr 9;79(5):154. doi: 10.1007/s00284-022-02848-y (PMC8994737; doi:10.1007/s00284-022-02848-y)

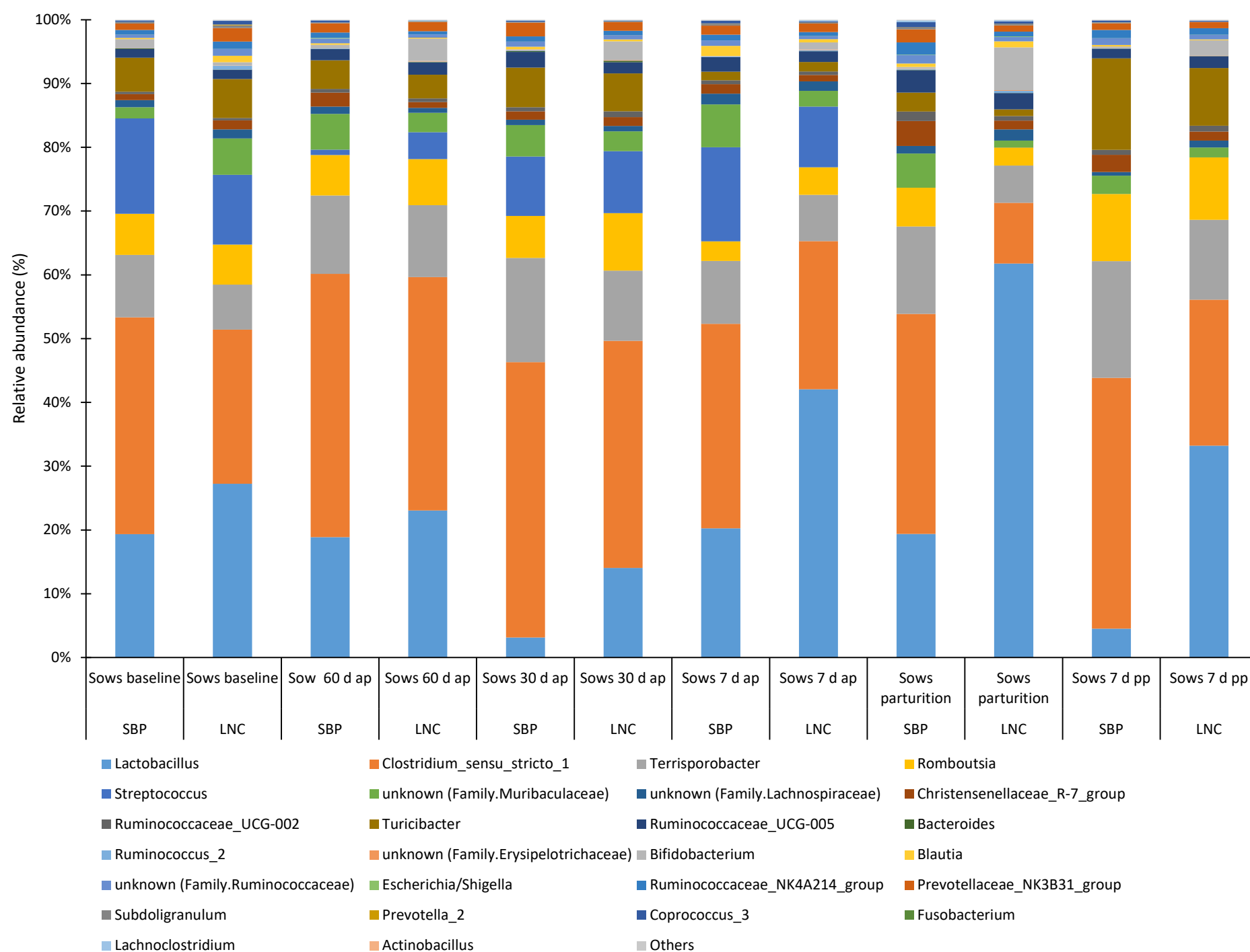

Supplement: Supplementary file 1 — Supplementary file1 (PDF 49 kb) [file 284_2022_2848_MOESM1_ESM.pdf]

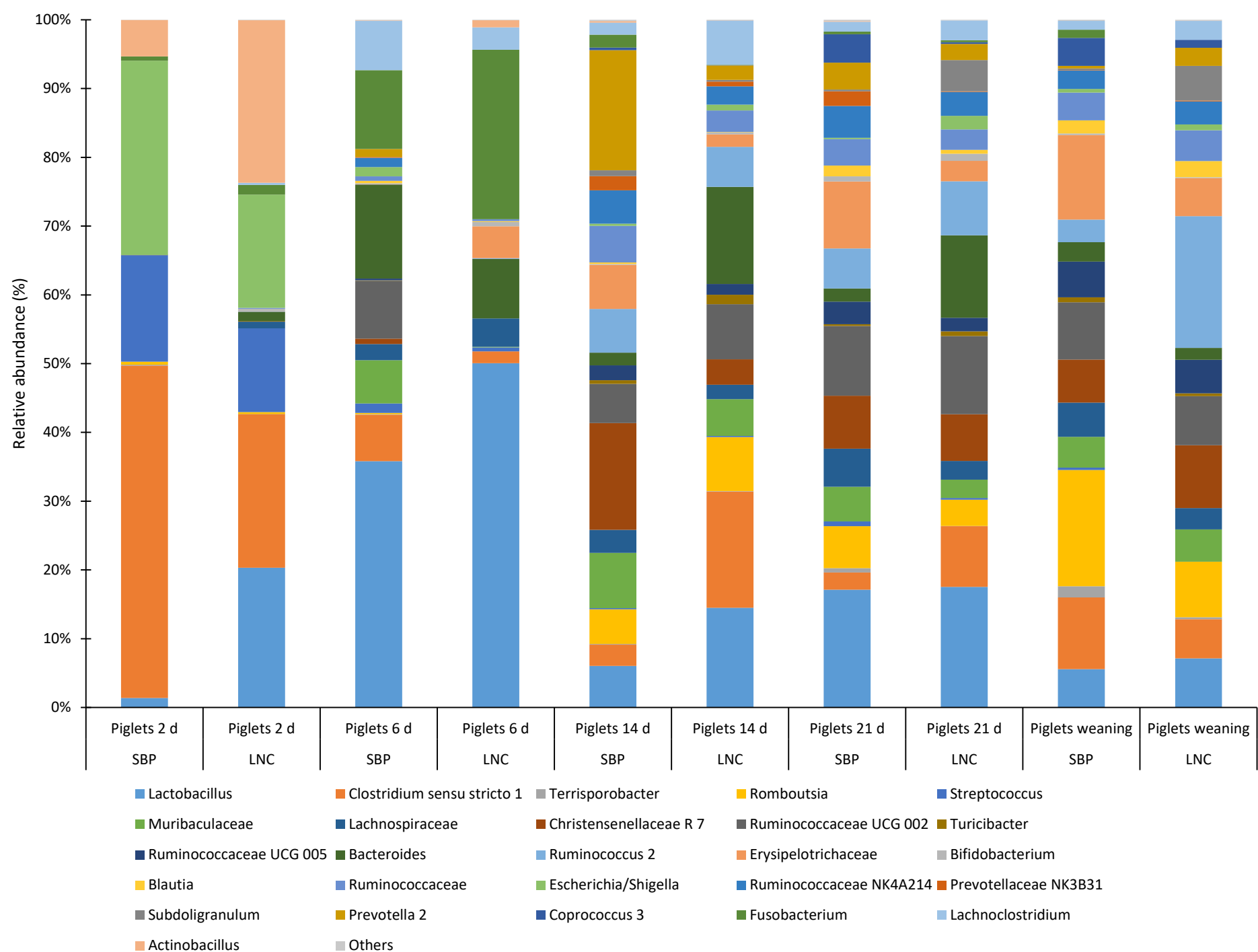

Supplement: Supplementary file 2 — Supplementary file2 (PDF 48 kb) [file 284_2022_2848_MOESM2_ESM.pdf]
